# Clonal Evolution of a Case of Treatment Refractory Maxillary Sinus Carcinoma

**DOI:** 10.1371/journal.pone.0045614

**Published:** 2012-09-28

**Authors:** Shilpi Arora, Ronald L. Korn, Elizabeth Lenkiewicz, Irene Cherni, Thomas G. Beach, Galen Hostetter, Michael T. Barrett, Glen J. Weiss

**Affiliations:** 1 The Translational Genomics Research Institute, Phoenix, Arizona, United States of America; 2 Scottsdale Medical Imaging, LLC, Scottsdale, Arizona, United States of America; 3 Sun Health Research Institute at Banner Healthcare, Phoenix, Arizona, United States of America; 4 Virginia G. Piper Cancer Center Clinical Trials at Scottsdale Healthcare, Scottsdale, Arizona, United States of America; B.C. Cancer Agency, Canada

## Abstract

**Background:**

Maxillary sinus carcinoma (MSC) is a rare cancer of the head and neck region. Patients are treated with surgery, radiation therapy, and chemotherapy and the treatment regimen is based on patient’s age, general health condition, disease stage, and its extent of spread. There is very little information available on the genetics of this disease. DNA content based flow sorting of tumor cells followed by array comparative genomic hybridization allows for high definition global assessment of distinct clonal changes within tumor populations.

**Methods:**

We applied this technique to primary and metastatic samples collected from a patient with radio- and chemotherapy refractory maxillary sinus carcinoma to gauge the progression of this disease.

**Results:**

A clonal *KIT* amplicon was present in aneuploid populations sorted from the primary tumor and in divergent subclones arising in metastatic foci found in the brain, lung, and jejunum. The evolution of these subclones was associated with distinct genetic aberrations and DNA ploidies.

**Conclusion:**

The information presented here paves the path to understanding the development and progression of this disease.

## Introduction

Maxillary sinus carcinoma (MSC) is an exceedingly rare cancer and there are no established guidelines on how best to treat advanced cases. Annually, these comprise approximately 3% of all head and neck carcinomas [1], or 1,100 cases, with up to 25% developing distant metastases [2]. Risk factors for the disease include nickel dust, mustard gas, isopropyl oil, chromium, or dichlorodiethyl sulfide. Wood dust exposure also increases the risk. Some of these products are found in furniture-making businesses, leather and textile industries [3], [4]. Viral infection, radiation exposure, and smoking have also been associated with the disease [5].

For localized or locally advanced disease, treatment involves primary surgery, radiation, or a combination of the two [1], [6]. When the disease relapses and is neither amenable to surgery nor radiation therapy, palliative systemic chemotherapy may be administered.

Previously published literature on MSC is limited but recently several groups have published insights into this rare cancer. Lopez *et al.* utilized aCGH and determined the most common regions of gains and losses in MSCs [7]. Some of these identified genomic changes were similar to head and neck squamous cell carcinomas (HNSCCs), even though unlike HNSCCs, smoking and alcohol use are not the most common etiologic factors for MSCs. *TP53* and *KRAS* mutation analysis have also been reported, but relatively fewer genome-wide studies have been performed in these cancers [3], [8], [9]. Thus, to evaluate clonal evolution of this rare cancer, we examined the initial surgical resection sample along with several tumors collected as part of a rapid warm autopsy program in a patient who developed radio- and chemotherapy refractory MSC disease.

One of the challenges to study somatic genetics of human cancer *in vivo* is the presence of admixtures of genomically normal cells in patient tumor samples. These can dilute the presence of aberrations such as homozygous deletions and interfere with the mapping of amplicons and their boundaries in biopsies of interest. For example, even 5% or less normal cell contamination can obscure the detection of homozygous deletions in samples of interest [10]. Furthermore, tumor biopsies frequently contain multiple neoplastic populations that cannot be distinguished by morphology [11]. Consequently, it is difficult to distinguish whether aberrations in a sample of interest are concurrent in a single clonal population that together may represent a unique prognostic marker or therapeutic vulnerability in a cancer, or if they occur in distinct populations in the same sample. For example, studies of several other cancer types including the cancers of the breast, colorectal and brain that surveyed somatic mutations in over 18,000 genes showed ∼80 gene-specific mutations in each cancer type [12]. Few highly recurrent mutations were detected; the majority of mutations occurred with a prevalence of <5% with little overlap between cancers. These and several other reports definitely challenge the concept of collective cancer genomes [13], [14], [15]. Thus, there is a need to not only isolate tumor from normal cells (which, most scientists have been doing by laser capture microdissection of the tumor specimens), but to also enrich clonal neoplastic cells in order to apply high definition genomics to study the clinical behaviors of cancer in patients *in vivo*. To study the genomic basis of the progression of metastatic MSC we used DNA content based flow cytometry to isolate the nuclei of tumor cells from a series of biopsies obtained from this rapid autopsy [16], [17]. Each sorted sample was interrogated with array comparative genomic hybridization (aCGH). Our results provide a unique description of the clonal evolution of aneuploid tumor populations with a common c-KIT amplicon during the progression to metastatic MSC.

## Materials and Methods

### Case Report

**Figure 1:**
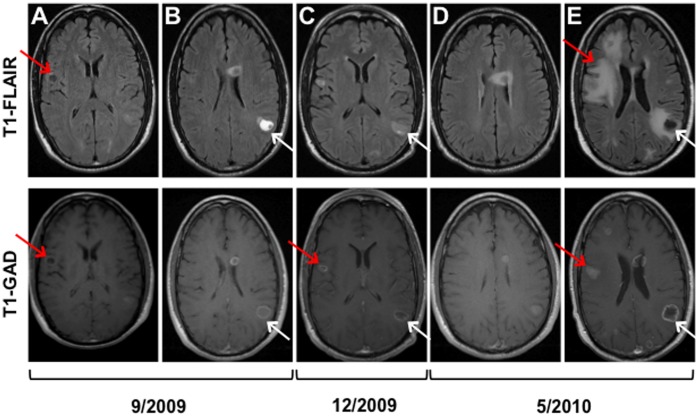
Brain (1.5T MRI appearance of multiple brain metastasis over time. Top row contains axial images from the T1-FLAIR (Flip Angle Inversion Recovery) sequences. Bottom row displays corresponding axial contrast-enhanced (T1-GAD) images of selected metastasis that were the target of rapid autopsy analysis. Notice that the right precentral gyrus brain metastasis evolved into a more solid appearing lesion over time (panels A, C and E bottom row, red arrow) and incited significant white matter edema on the final scan (panel E, top row, red arrow). The left inferior parietal lesion (panels B, C and E panels-white arrows) evolved to become more necrotic over time with mild incitement of white matter edema on T1-FLAIR on the final image (panel E, top row, white arrow) and an enhancing rind of tumor surround the necrotic portion of the lesion (panel E, bottom row-white arrow). Such behavior on MRI might be anticipated given the distinct clonal nature of each of the tumor metastasis.

Tissues were obtained with approval of the local institutional review board (Scottsdale Healthcare, Scottsdale, AZ [SHC]) and written consent from the patient’s next of kin. The patient, a 46-year-old never-smoker, working as a photography editor presented with right side facial tingling. His only other relevant history was a prior resected basal cell carcinoma (BCC) six years earlier. He was diagnosed with localized MSC and underwent a right maxillectomy at another institution in June 2006. After resection, the pathology specimen revealed a 2.8 cm undifferentiated MSC (histologically distinct from BCC) with clear margins and the disease was classified as T3N0M0 (stage III). This was followed by adjuvant image-guided radiation therapy (IMRT) from August to September 2006 to the right maxillary duct/orbit to a total dose of 6,400 cGy. However, his tumor relapsed with a solitary metastasis to the right upper lung in September 2007 (**Figure S1A**). Biopsy of his right maxillary sinus was negative for tumor and MRI of the brain was negative for recurrence. Thus, with only a solitary metastasis, the patient underwent video-assisted thoracoscopic surgery (VATS) wedge resection at a different institution with confirmation of metastatic MSC measuring 1.3 cm. On follow-up imaging, there was interval development of a non-calcified 0.9 cm nodule in the left lower lobe of the lung on CT scan in December 2007 (**Figure S1B**), that grew larger than 1 cm and was 18F-fluorodeoxyglucose positron emission tomography (PET) avid in February 2008. Because of the short interval from metastatectomy to development of another metastasis and the rarity of his cancer, the patient was referred to SHC for systemic therapy on a clinical trial beginning in March 2008. As seen in **Figure S2A**, the left lower lobe mass measured 2.0 cm and was the only site of disease on imaging. He was treated with a novel topoisomerase I (TOP1) inhibitor, achieving a partial response (tumor mass decreased to 0.7 cm in this sole site of measurable disease by RECIST criteria [18] (**Figure S2B**). By the end of 2008, this site of disease progressed and remained the sole site of evaluable and measurable disease, and the patient was withdrawn from the clinical trial. The patient opted for definitive radiotherapy over surgical resection and IMRT to the left lower lobe lesion now measuring 1.0 cm was administered from March to April 2009 to a total dose of 6,000 cGy. Unfortunately, in September 2009, this lung tumor had grown to 2.1 cm (**Figure S2C**) and multifocal newly identified brain metastases were visualized on magnetic resonance imaging (MRI) up to 1.8 cm in size [**representative examples in** **Figure 1A** **(top/bottom)- 1B (top/bottom)**]. The left lower lobe lung tumor was biopsied at this time, confirming metastatic undifferentiated MSC. The specimen was sent for commercial immunohistochemistry (IHC) and gene microarray testing [19], [20] that revealed several potentially druggable targets (**Table S1**). The patient was treated with whole brain radiation therapy (WBRT). However, at follow-up MRI in December 2009, while there was diminished enhancement of a left occipital lobe metastasis, the lesion had grown from 1.3 to 2.0 cm in contrast to decrease in the size of the other brain metastases (**representative examples in** **Figure 1C** **)**. Thus, stereotactic radiosurgery was delivered in December 2009 to a total dose of 2,500 cGY in five fractions. Additionally, the left lower lung mass was also larger at 4.2 cm, and there was a new mass identified in the left kidney (**Figure S2D and S3A–C**). Despite the tempo of disease progression, the patient remained in excellent performance status and had minimal visual symptoms. He was then initiated on irinotecan and sorafenib chemotherapy based on TOP1 and KIT expression by IHC (**Table S1**). His treatment was complicated by severe diarrhea and dehydration resulting in hospitalization. In May 2010, the patient had a grand mal seizure and MRI revealed new and progressive brain metastases (representative tumor images in **Figure 1D–E** **and Figures S4, S5, S6, and S7**). With his performance status significantly diminished, patient enrolled in hospice and a rapid warm autopsy was performed within four hours of his death.

## Methods

### Flow Sorting and Array CGH

All samples were stored in −80°C. Flow sorting of aneuploid and diploid populations was performed for each sample using established DNA content-based protocols for neoplasias [16], [17]. Briefly, biopsies were minced in the presence of NST buffer [146 mM NaCl buffer containing 10 mM Tris-HCl (pH 7.5), 0.2% Nonidet P40] and DAPI. Nuclei were disaggregated, filtered, and flow sorted with an Influx cytometer (Becton-Dickinson) with UV excitation and DAPI emission collected at >450 nm. DNA content and cell cycle were analyzed using the MultiCycle software program (Phoenix Flow Systems), San Diego, CA) [21]. For each sample, ploidy and cell cycle fractions (G1, S, G2/M) was collected.

Flow-sorted tumor cell populations were profiled using Agilent 1 M 60mer oligonucleotide CGH arrays. Briefly, DNA was extracted from each sample using a QIAmp DNA Micro kit (Qiagen; Valencia, CA). A 100 ng aliquot of genomic DNA from each sample was amplified using the GenomiPhi amplification kit (G.E. Healthcare; Piscataway, NJ). For each hybridization, 1 ug of amplified sample and 1 ug of amplified commercial pooled 46,XX reference template was digested with DNase I then labeled with Cy-5 dUTP and Cy-3 dUTP respectively, using a BioPrime labeling kit (Invitrogen; Carlsbad, CA). Labeling reactions, hybridizations, and analyses were performed as previously described in Ruiz *et al*, 2011 [17].

### Immunohistochemistry (IHC)

A small piece of all the tissues obtained after warm autopsy was formalin-fixed and paraffin-embedded. In addition, paraffin blocks from other samples from the same patient were also included. H & E staining was performed on tissue sections from all the blocks. Each block was sectioned at 5 µM and affixed to Fisher brand Superfrost® plus slides (Fisher Scientific) via water flotation and overnight drying. Slides were deparaffinized with xylene, rehydrated through a series of graded ethanol baths and antigen retrieved on-line using a BondMax™ autostainer (Leica Microsystems, INC Bannockburn, IL). Staining for c-KIT was achieved using rabbit polyclonal KIT antibody (DAKO, PA) and visualized using the Bond™ Polymer Refine Detection kit (Leica) using 3,3′-diaminobenzidine tetrahydrochloride chromogen as substrate. IHC scoring was performed on the basis of extensiveness and intensity. Stained tissues were scored using general intensity (value of 0, 1, 2, or 3). Positive macrophage/negative tissue staining was found across all markers and served as internal controls across the protocol. Examples of staining are shown in **Figure S8**.

### PCR

To validate the aCGH findings, qPCR analysis of PKP4 and miR-651 genomic regions, as well as neighboring genomic regions were performed using SYBR green chemistry (Roche, NJ) according to the manufacturer’s instructions. Briefly, flow-sorted and phi29-amplified DNA was subjected to a PCR composed of 35 cycles with an annealing temperature of 59°C. The sequences of the primers used are listed in **Table S2**. Actin was used as a reference. Data was analyzed by delta-delta Ct method.

## Results

### Aneuploid Population and aCGH

**Figure 2:**
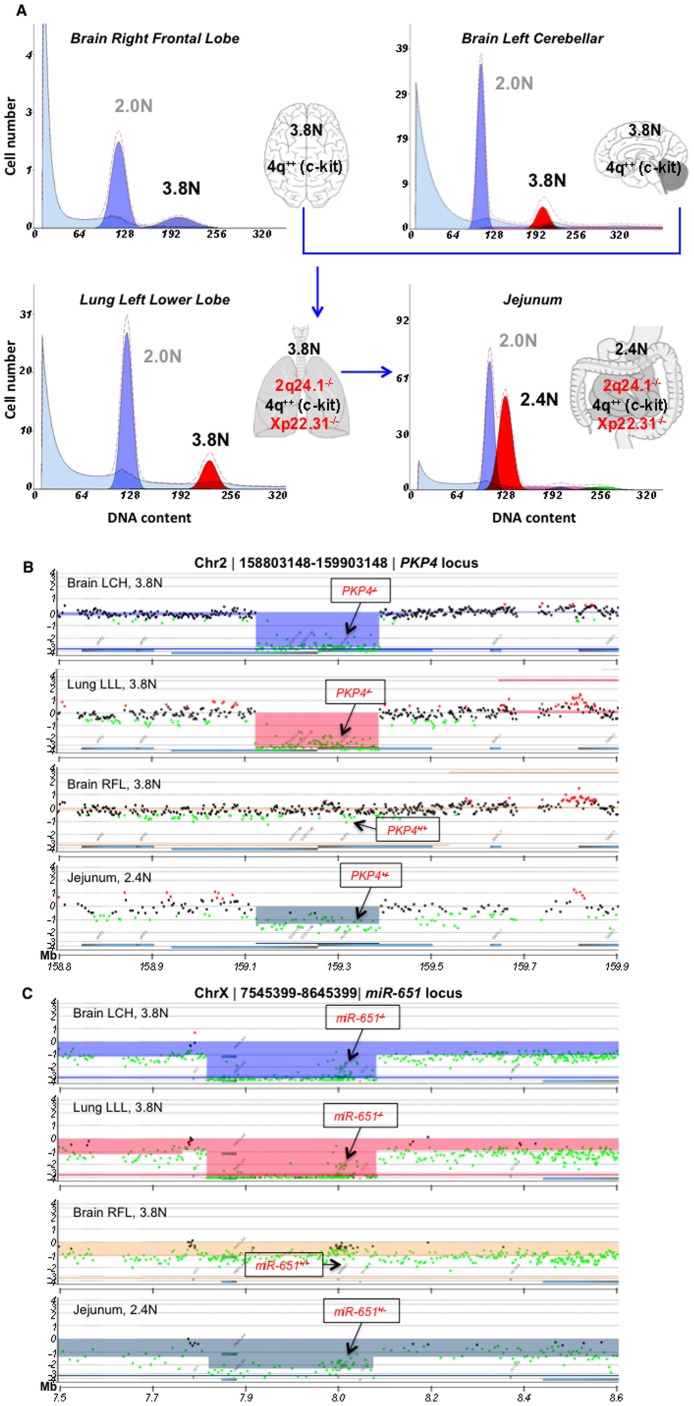
Clonal analyses of the autopsy samples from the maxillary sinus cancer patient. A) DAPI-based DNA content analysis detected a 3.8N clonal population in brain right frontal pole brain left cerebellar and lung left lower lobe samples, while a 2.4N clonal population was seen in the jejunum sample. The diploid and aneuploid populations were sorted for subsequent aCGH studies. **B**) Zoomed in chromosome view showing the *PKP4* gene locus in the above four samples. **C**) Zoomed in chromosome view showing the *pre-miR-651 (labeled miR-651)* gene locus status in the above four samples.

Because our patient's tumor resumed growth despite several courses of systemic chemotherapy and radiation therapy, we speculated that acquired secondary genetic changes evolved with the evolution of resistance to these therapies. We first examined the aneuploid population of several metastatic foci [brain right frontal lobe (BRFL), brain left cerebellum (BLC), lung left lower lobe (Lung LLL), and jejunum] collected at the time of autopsy. We detected several aberrations, including amplification of a region on chromosome 4q, which includes *KIT,* in all four flow sorted tumor specimens. There were additional deleted regions present in the metastases including homozygous deletions (aCGH log_2_ ratio< −3.0) of the *PKP4* gene (2q24.1) and *pre-miR-651* (Xp22.31) in at least one of the metastatic sites. In addition the tumor population sorted from the jejunum metastastic locus had a unique ploidy (2.4N *vs* 3.8N in all the other three specimens), suggesting at least three distinct clonal populations (**Figure 2A–C** **and** **Table 1**). The patient’s primary tumor was FFPE and several attempts to flow sort were performed. Unfortunately the sample was too degraded for this type of analysis and hence, only PCR validation of targets and IHC could be performed.

**Table 1:** Clinicopathological and molecular details for the samples from the MSC patient.

Age	Gender	Tissue types	KIT IHC	Flow sorted CGH result	Validated clonal population
45	M	Undifferentiated carcinoma-2006 sample from initial surgery	3+	n/a	4q amplification
Autopsy samples, May 2010		Left perinephric fat	3+	n/a	4q amplification, Xp22.31 deletion
		Left pontine tegmentum	3+	n/a	4q amplification, Xp22.31 deletion
				
		Left lateral occipital lobe	3+	n/a	4q amplification, Xp22.31 deletion
		Left inferior parietal lobe	3+	n/a	4q amplification, Xp22.31 deletion
		Left cerebellar hemisphere	3+	4q amplification	4q amplification, Xp22.31 deletion
				
		Cerebellar leptomeninges	3+	n/a	4q amplification, Xp22.31 deletion
		Pancreas	3+	n/a	4q amplification, Xp22.31 deletion
				
		Jejunum	3+	4q amplification, 2q24.1 deletion, Xp22.31 deletion	4q amplification, Xp22.31 deletion
		Left periventricular white matter at anterior horn lateral ventricle	3+	n/a	4q amplification, Xp22.31 deletion
		Left kidney	3+	n/a	4q amplification, Xp22.31 deletion
		Left lower lobe lung	3+	4q amplification, 2q24.1 deletion, Xp22.31 deletion	4q amplification, 2q24.1 deletion, Xp22.31 deletion
		Right frontal pole	n/a	4q amplification	No deletion of 2q24.1 or Xp22.31

### Validation of Results

**Figure 3:**
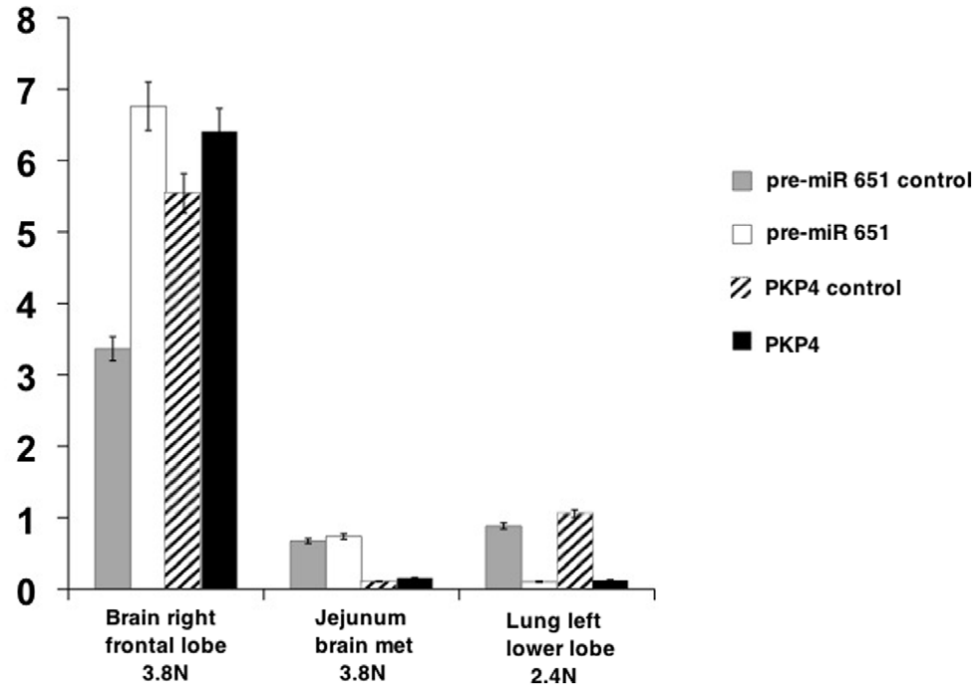
The aCGH results were validated by qPCR using the flow-sorted DNA. Primers were designed for *PKP4* and *pre-miR-651* genomic sequences. Primers were also designed using sequences outside the deleted regions for *PKP4* and *pre-miR-651* and were called *PKP4-*control and *pre-miR-651-*control respectively. Actin was used as the universal control. qPCR was performed and fold change was calculated and plotted. The left lower lung tumor sample validated as expected for *pre-miR-651* and *PKP4* deletion and the brain right frontal lobe sample did not show any of these deletions validating the aCGH results. The jejunum sample did not validate for these results.

To validate these findings, we performed IHC for KIT expression (**Figure S8 and** **Table 1**) and qPCR on aneuploid DNA for *PKP4* and *pre-miR-651* regions, using diploid DNA as a control. Validation of these aberrations to determine clonality were assessed in the tumors that were flow sorted, the original surgical resection specimen from 2006, and additional samples collected at the time of autopsy. All available specimens from the presented case revealed strong KIT protein expression as determined by immunohistochemistry (**Figure S8**). The *KIT* amplification explains the over-expression of KIT in each of the metastatic lesions from the patient. The aCGH results were validated by qPCR using the flow sorted DNA. The Lung LLL sample validated as expected for pre-miR-651 and PKP4 homozygous deletions and the BRFL sample did not show either of these deletions as was observed in the aCGH results. In contrast the partial losses at these two sites observed in the 2.4N jejunum sample were not detected in our qPCR assays (**Figure 3**).

Interestingly, brain MRI images from September 2009, show that the right precentral gyrus tumor (probable *KIT* amplified clone) exhibited a different intensity and enhancement pattern compared to left inferior parietal tumor (*KIT* amplified, pre-miR-651 deletion clone) (**Figure 1**). The appearance of these lesions on subsequent MRIs evolved either towards a more solid appearance with marked incitement of white matter edema (right precentral gyrus tumor) or more necrotic appearance (left inferior parietal tumor) with less edema despite WBRT. Other brain metastases with pre-miR-651 deletions showed a similar appearance with surrounding edema similar to the left inferior parietal lobe tumor on MRI images preceding WBRT (data not shown).

## Discussion

Maxillary sinus cancer has relatively poor prognosis with five year survival rates at less than 50% [22]. Lymph node metastasis, advanced T stage, squamous cell histology, and positive surgical margins are classical known poor prognostic factors for MSC. There have been several recent reports on the microRNA, aCGH, and gene expression profiles of MSC patients [7], [8], [9], [23]. But, this is the first report to study the clonal populations of MSC arising in longitudinal samples from the same patient. We performed aCGH analysis on four samples from different metastasis sites that were collected after a rapid autopsy. The samples were first flow-sorted based on DNA content to identify then isolate pure populations of tumor cells for clonal analysis. The flow cytometry and aCGH results from these samples were compared to the primary tumor sample collected 4 years earlier at a different hospital. The results were also validated in a series of other samples collected after autopsy from the same patient. The patient was treated several times with radiotherapy and also with chemotherapy (including those based on the IHC findings) and the tumor was refractory to the treatment. One of the aims of this study was to closely follow disease progression and the clonally evolving metastases for molecular profiling and accumulation of data for future use in development of personalized treatment.

Upon flow sorting, we observed a 3.8N clonal population in 3 of the 4 metastatic sites namely, BRFL, BLC, and Lung LLL. The fourth metastatic site, jejunum showed a different aneuploid population (2.4 N) suggesting that this was another distinct clone that arose during disease progression. Amplification of the *KIT* genomic region was observed in all four samples, while homozygous loss (log_2_ratio <−3.0) of 2q24.1 and Xp22.31 was observed in the 3.8N tumor populations present in the BLC and Lung LLL suggesting that these clones were further evolved from the BRFL clone. This was an interesting finding in light of the disease progression in the patient because LLL metastasis was detected earlier than the BLC clone and remained the only site of metastasis for several months before the other sites were detected. These results show that molecular analyses of patient samples can add to the information about the tumor and help us in tracking back the progression of the disease. We validated the results and designed genomic primers for *PKP4* (in the 2q24.1 region) and pre-miR-651 (in the Xp22.31 region). We were able to validate the loss of *PKP4* and *pre-miR-651* in the BRFL and Lung LLL samples (we did not have enough sorted material for the BLC sample and hence those results could not be validated). Our aCGH data suggested that the distinct 2.4N population present in the jejunum had partial losses at both of the regions, however we were unable to confirm these results in our qPCR assays.

Loss of *pre-miR-651* has not been previously reported in the development or progression of cancer. *PKP4* belongs to a family of plakophilins, which are members of the armadillo multigene family. Armadillo-related proteins function in both cell adhesion and signal transduction, and also play a central role in tumorigenesis. Interestingly, there is one report by Papagerakis *et al*. demonstrating that oropharyngeal cancers there is an inverse correlation between tumor size and PKP4 protein expression, thus the patients with low expression of PKP4 have larger tumor size [24]. This observation is in accordance with our observation that loss of PKP4 associated with tumor progression. *Pre-miR-651* has not been previously studied. According to the miRNA binding site prediction on microrna.org http://www.microrna.org/microrna/getTargets.do?matureName=hsa-miR-651&organism=9606, several genes important in cancer development and progression are predicted to be targets of *pre-miR-651* but these targets will need to be validated before we make any strong predictions on the role of *pre-miR-651* in cancer progression and metastasis.

In addition, radiological analysis of the brain MRI images showed that the metastatic lesions had different characteristics over time that could be classified loosely either as an evolving solid lesion with marked white matter edema (right prefrontal gyrus tumor) or necrotic lesion with little to mild incitement of white matter edema (left inferior parietal tumor). These observations suggest that the different clonal populations of tumor exhibit different imaging characteristics. Such linkage between imaging and genomic expression has been noted in other tumors, e.g. gliobastoma multiforme [25]. There is clearly room for improvement in the treatment of advanced MSC. These observations need to be validated in other MSC patient samples to see whether these genetic aberrations are a common occurrence in MSC patients or whether MSC development is more heterogeneous in nature. Thus, utilization of this novel modality may facilitate identification of agents that may provide therapeutic benefit to patients with advanced MSC. Identification of selected clonal aberrations and the biological processes they regulate arising in primary MSC tumors that indicate a high risk of recurrence and metastasis will advance individualizing therapy and improve the outcome of patients with rare cancers. The ability to characterize clonal evolution of this rare cancer and identify its Achilles’ heel can significantly impact treatment, leading to more personalized medicine.

## Supporting Information

Figure S1 (A) Blue arrow points to tumor in the right upper lobe seen on CT scan on September 2007 (B) Blue arrow points to tumor in the left lower lobe of the lung seen on CT scan on December 2007.
(PPT)

Figure S2 **Lung metastasis seen on contrast enhanced CT (lung windows) at indicated dates prior to rapid autopsy.** Note the initial decrease in lesion size following radiation therapy followed by lobulated enlargement with time and obliteration of adjacent bronchioles.
(PPTX)

Figure S3 **Renal metastasis seen on contrast enhanced multi slice CT scan obtained during the nephrogenic phase of enhancement at different times before autopsy.** The metastatic lesion demonstrated an infiltrative appearance atypical for solid tumor renal metastasis.
(PPTX)

Figure S4 **Cerebellar and pontine metastatic lesions on axial MRI brain scans obtained at the dates indicated prior to rapid autopsy.** T1weighted flip angle inverted recovery sequence (FLAIR) shows the development of diffuse high signal surround the primary lesion indicative of peritumoral edema (red arrow). T1 weighted gadolinium (Gad) enhanced images (blue arrows) shows the progressive peripheral (ring) enhancement of the targeted biopsy lesion over time, a finding due to increasing disruption of the blood-brain barrier. Note the similar behavior of the other metastatic lesion in the pons (yellow arrow).
(PPTX)

Figure S5 **Left lateral occipital lobe metastasis on axial MRI brain scans obtained at the dates indicated prior to rapid autopsy.**
(PPTX)

Figure S6 **On axial MRI brain scans obtained at the dates indicated prior to rapid autopsy.** T1weighted flip angle inverted recovery sequence (FLAIR) shows disappearance of necrosis from an earlier date on the 9/2009 scan towards the development of diffuse high signal surrounding the primary lesion indicative of peritumoral edema (red arrow). T1 weighted Gad enhanced images (blue arrows) shows the progressive peripheral (ring) enhancement of the targeted biopsy lesion over time, a finding due to increasing disruption of the blood-brain barrier. Note the similar behavior of the other metastatic lesion in the frontal lobes on T1-Gad sequences.
(PPTX)

Figure S7 **Right frontal pole (red arrows) and left periventricular (blue arrows) metastasis seen on axial MRI brain scans obtained at the dates indicated prior to rapid autopsy.**
(PPTX)

Figure S8 **KIT immunohistochemical staining (A) example of 0+ (negative) staining on pleomorphic sarcoma tumor tissue, (B) example of 3+ staining of MSC tumor from the cerebellar leptomeninges.**
(PPTX)

Table S1 (DOC)

Table S2 (XLS)
