# Supplementary figures and images for: Clonal Evolution of a Case of Treatment Refractory Maxillary Sinus Carcinoma
Source: PLoS One. 2012 Sep 28;7(9):e45614. doi: 10.1371/journal.pone.0045614 (PMC3460998; doi:10.1371/journal.pone.0045614)

## Slide 1
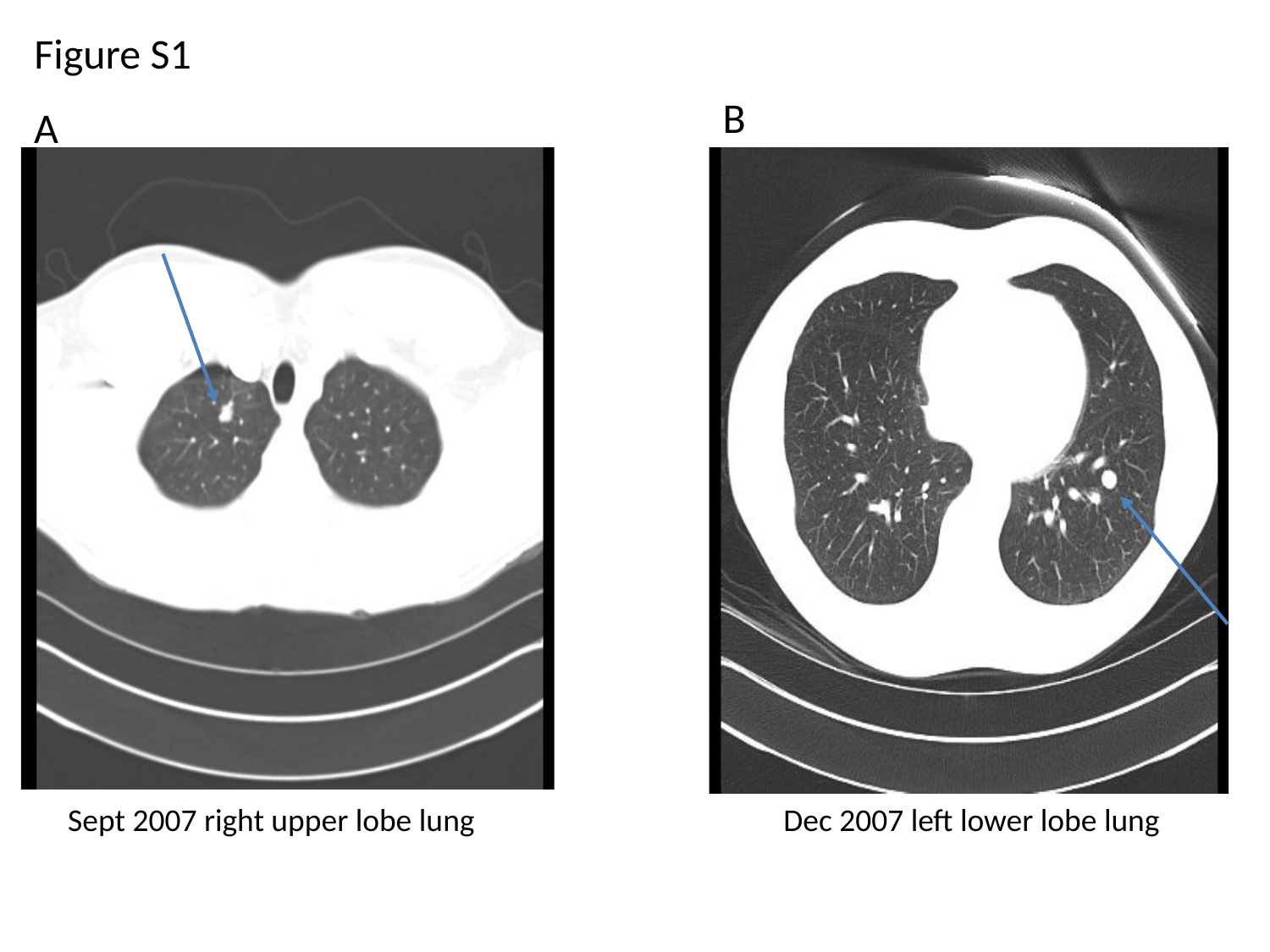

Figure S1
B
A
Sept 2007 right upper lobe lung
Dec 2007 left lower lobe lung

Supplement: Figure S1 — (A) Blue arrow points to tumor in the right upper lobe seen on CT scan on September 2007 (B) Blue arrow points to tumor in the left lower lobe of the lung seen on CT scan on December 2007. (PPT) [file pone.0045614.s001.ppt]
