# Supplementary material for: Clonal Evolution of a Case of Treatment Refractory Maxillary Sinus Carcinoma
Source: PLoS One. 2012 Sep 28;7(9):e45614. doi: 10.1371/journal.pone.0045614 (PMC3460998; doi:10.1371/journal.pone.0045614)
Supplement: Table S1 — (DOC) [file pone.0045614.s009.doc]

**Table S1. CLIA-certified laboratory IHC and gene expression profiling results**

| **Gene** | **IHC Intensity (% stained)** | **mRNA Expression Fold change*** | **Potential Drug** |
| --- | --- | --- | --- |
| KIT | 3+ (95) | 146 | Imatinib |
| TOP1 | 2+ (70) | 0.88 | Irinotecan |
| PTEN | 0 | 1.20 | N/A |
| ERCC1 | 0 | 0.49 | N/A |
| ER | 0 | N/A | N/A |
| PR | 0 | N/A | N/A |
| SPARC | 0 | 0.83 | N/A |
| HER2 | 0 | N/A | N/A |
| BCRP | 2+ (70) | N/A | N/A |
| MRP1 | 2+ (50) | N/A | N/A |
| RRM1 | 2+ (50) | 3.15 | N/A |
| MGMT | 2+ (50) | 0.56 | N/A |
| TS | 2+ (25) | 43.32 | N/A |
| TOP2A | 2+ (20) | 49.13 | N/A |
| PGP | 1+ (80) | N/A | N/A |
| *Relative to control tissue; N/A not applicable | | | |
